# Improved DNA Extraction and Amplification Strategy for 16S rRNA Gene Amplicon-Based Microbiome Studies

**DOI:** 10.3390/ijms25052966

**Published:** 2024-03-04

**Authors:** Bo-Young Hong, Mark Driscoll, Dawn Gratalo, Thomas Jarvie, George M. Weinstock

**Affiliations:** 1 Dr. Kiran C. Patel College of Allopathic Medicine, Nova Southeastern University, Fort Lauderdale, FL 33328, USA; 2 Jackson Laboratory for Genomic Medicine, Farmington, CT 06032, USA; 3 Intus Biosciences, Farmington, CT 06032, USA

**Keywords:** human microbiome, 16S rRNA gene sequencing

## Abstract

Next-generation sequencing technology has driven the rapid advancement of human microbiome studies by enabling community-level sequence profiling of microbiomes. Although all microbiome sequencing methods depend on recovering the DNA from a sample as a first critical step, lysis methods can be a major determinant of microbiome profile bias. Gentle enzyme-based DNA preparation methods preserve DNA quality but can bias the results by failing to open difficult-to-lyse bacteria. Mechanical methods like bead beating can also bias DNA recovery because the mechanical energy required to break tougher cell walls may shear the DNA of the more easily lysed microbes, and shearing can vary depending on the time and intensity of beating, influencing reproducibility. We introduce a non-mechanical, non-enzymatic, novel rapid microbial DNA extraction procedure suitable for 16S rRNA gene-based microbiome profiling applications that eliminates bead beating. The simultaneous application of alkaline, heat, and detergent (‘Rapid’ protocol) to milligram quantity samples provided consistent representation across the population of difficult and easily lysed bacteria equal to or better than existing protocols, producing sufficient high-quality DNA for full-length 16S rRNA gene PCR. The novel ‘Rapid’ method was evaluated using mock bacterial communities containing both difficult and easily lysed bacteria. Human fecal sample testing compared the novel Rapid method with a standard Human Microbiome Project (HMP) protocol for samples from lung cancer patients and controls. DNA recovered from both methods was analyzed using 16S rRNA gene sequencing of the V1V3 and V4 regions on the Illumina platform and the V1V9 region on the PacBio platform. Our findings indicate that the ‘Rapid’ protocol consistently yielded higher levels of Firmicutes species, which reflected the profile of the bacterial community structure more accurately, which was confirmed by mock community evaluation. The novel ‘Rapid’ DNA lysis protocol reduces population bias common to bead beating and enzymatic lysis methods, presenting opportunities for improved microbial community profiling, combined with the reduction in sample input to 10 milligrams or less, and it enables rapid transfer and simultaneous lysis of 96 samples in a standard plate format. This results in a 20-fold reduction in sample handling time and an overall 2-fold time advantage when compared to widely used commercial methods. We conclude that the novel ‘Rapid’ DNA extraction protocol offers a reliable alternative for preparing fecal specimens for 16S rRNA gene amplicon sequencing.

## 1. Introduction

The advent of Next-Generation Sequencing (NGS) technology has enabled rapid, cost-effective microbiome profiling that can be used to study the impact of microbial community structure on health and disease. Sequence-based profiling of microbiomes, whether using 16S rRNA gene amplicons or shotgun methodology, depends on a lysis and DNA isolation method that is stringent enough to lyse all cells while not severely damaging the DNA. It is well established that lysis methods can be a major determinant of microbiome profile bias in studies performed with stool [1,2,3,4,5,6,7] and oral samples [8,9,10]. In order to obtain an unbiased representation of heterogeneous bacterial communities within a sample, the lysis method employed must be robust enough to lyse all microbes while at the same time preserving DNA quality. Gram-positive Firmicutes species tend to contain thicker cell walls, which makes them more difficult to lyse, and it is known that Firmicutes are under-represented by the HMP protocol. Gentle lysis methods will tend to under-represent Gram-positive bacteria, while more stringent bead-beating methods are difficult to automate and can introduce variability because they can rapidly damage the DNA of easily lysed microbes. The intensity of bead beating can vary depending on the position of a sample in the bead beater instrument, or because of minor variations in tube placement, as well as variations in bead beater duration and intensity. Gram-positive bacteria tend to have thicker cell walls with several layers of peptidoglycan that can vary in chemical structure [11], biasing enzymatic/lysozyme lysis methods. The major structural differences between bacteria provide significant challenges to the development of a DNA extraction method with uniform lysis properties across complex bacterial communities.

We developed and tested a non-bead beating, non-enzymatic, novel ‘Rapid’ microbiome DNA extraction procedure suitable for 16S rRNA gene-based microbiome profiling applications. The ‘Rapid’ protocol was designed for uniform lysis of diverse populations of microbial cells, including difficult-to-lyse bacteria, by applying a unique, single alkaline/heat/lysis buffer combination to sample volumes of a few milligrams, which simplifies sample handling while rapidly supplying a sufficient high-quality DNA yield for both short and long amplicon rRNA PCR applications.

Alkaline lysis methods disrupt the bacterial cell by denaturing and solubilizing membrane components. Since 1979 [12], alkaline lysis using sodium hydroxide (NaOH) has been widely used for isolation of plasmid DNA. In 2014, a potassium hydroxide (KOH) based DNA extraction method was shown to be effective in the target lysis of the Gram-positive genera *Bacillus*, *Streptomyces*, *Micromonospora*, *Nonomuraea*, *Microbispora*, and *Staphylococcus* [13]. Unfortunately, KOH-based methods result in the rapid precipitation of commonly used lysis buffers, which is perhaps why KOH methods were not more widely used. Application of heat to precipitation-resistant lysis buffers in a basic KOH solution result in a simultaneous attack on the cell wall and membrane that may be more effective than independent treatments. Although alkaline protocols were previously demonstrated to effectively break down targeted Gram-positive complex cell walls, patent US 10,774,322 B2 [14] for the ‘Rapid’ technique was granted for this method because there was previously no report exploring the potential benefit of an alkaline KOH DNA extraction method combined with heat and lysis buffer mixtures in 16S rRNA gene amplicon-based microbiome studies targeting the entire bacterial community.

In the present study, we evaluated a novel alkaline-based DNA extraction method utilizing a mock community to test the true representation of bacterial communities against various other commercially available lysis protocols. We further evaluated alkaline-based DNA extraction methods using stool samples from 20 human subjects to demonstrate the effectiveness of this protocol for 16S rRNA sequencing-based microbiome studies. Bacterial 16S rRNA gene sequence data from both mock communities and human fecal microbiomes were generated as V1V3, V4 amplicons for MiSeq sequencing (Illumina, San Diego, CA, USA), and full-length 16S rRNA V1V9 amplicons for Sequel sequencing (Pacific Biosciences, Menlo Park, CA, USA) for the in-depth microbiome analyses and comparisons.

## 2. Methods

### 2.1. Commercial Mock Community—ZymoBIOMICS Microbial Community Standard 

The ZymoBIOMICS Microbial Community Standard containing three Gram-negative bacteria with relatively fragile cell walls, five Gram-positive bacteria with thicker cell walls, and two fungal species were tested. Theoretical composition based on genomic DNA consist of 12% *Listeria monocytogenes*, 12% *Pseudomonas aeruginosa*, 12% *Bacillus subtilis*, 12% *Escherichia coli*, 12% *Salmonella enterica*, 12% *Lactobacillus fermentum*, 12% *Enterococcus faecalis*, 12% *Staphylococcus aureus*, 2% *Saccharomyces cerevisiae*, and 2% *Cryptococcus neoformans*. Yeast genes were not included as part of the study and were absent from the microbiome profiles presented as part of this study.

### 2.2. Mock Community

A custom mock community consisting of 10 bacterial species was assembled (Supplementary Table S1). Bacterial species were selected to include members of the phyla Bacteroidetes, Firmicutes, Proteobacteria, and Actinobacteria, all of which are commonly found in the human gut. Primer specificity was another consideration. Gut bacterial species with 0 to 3 bases mismatched to the 27F forward primer was included in the mock community. Based on these criteria, *Lactobacillus paracasei* (isolate from our lab), *Enterococcus faecalis* ATCC^®^ 47077^TM^ OG1RF, *Escherichia coli* ATCC^®^ 700926^TM^MG1655, *Bifidobacterium dentium* ATCC^®^ 27678^TM^, *Bacteroides vulgatus* ATCC^®^ 8482^TM^, *Prevotella oralis* ATCC^®^ 33269^TM^, *Bacteroides caccae* ATCC^®^ 43185^TM^, *Prevotella copri* DSM18205, *Ruminococcus lactaris* ATCC^®^ 29176^TM^, and *Faecalibacterium prausnitzii* (isolate from our lab) were selected. The commercially available mock community tested using the V4 amplicon was obtained from Zymobiomics, containing *Pseudomonas aeruginosa, Escherichia coli*, *Salmonella enterica*, *Listeria monocytogenes*, *Bacillus subtilis*, *Lactobacillus fermentum*, *Staphylococcus aureus,* and *Enterococcus faecalis*. The mock also contained DNA from the eukaryotes *Saccharomyces cerevisiae* and *Cryptococcus neoformans.* To make the mock community, bacterial cells were freshly cultured and counted using a Neubauer Chamber hemocytometer and diluted to contain equal numbers of cells (10^7^ cells uL^−1^), and then normalized for 16S rRNA gene copy numbers (4~7) in order to include an equal number of 16S rRNA gene copies for each taxon. Normalized bacterial cell pools were centrifuged (5000× *g*), and pellets were used for 6 different DNA extraction methods.

### 2.3. Human Subject Fecal Sample Collection

Fecal samples from 20 subjects were included in this study. Subjects were enrolled at the Seoul National University, under an IRB-approved protocol. Fecal samples were collected from 10 hospitalized individuals undergoing treatment for lung cancer and 10 control subjects. Fecal samples were stored at −80 °C until processing. 

### 2.4. DNA Extraction

Six DNA extraction methods that employ a variety of lysis steps were evaluated. Methods included the novel Rapid protocol (chemical only), a new bead pasting protocol (mechanical only), and four commercially available protocols that included combinations of enzymatic, chemical, and mechanical methods, one of which was used as a standard protocol in the HMP (Qiagen PowerSoil kit). Two of the four commercial kits included enzyme-based lysis (Epicentre MasterPure and QiaAmp Stool), and two included mechanical bead beading (Qiagen PowerSoil kit and Zymo DNA/RNA Mini). Samples were purified in duplicate for each DNA extraction method. All six DNA extraction protocols were used to evaluate the custom mock community, which contains organisms with widely varying resistance to lysis. A Zymobiomics commercial mock community was processed using the Rapid protocol, and results were compared to the V4 amplicon results published by Zymobiomics. Human fecal samples were processed with the Rapid protocol and HMP protocols to determine whether the increased Firmicutes lysis seen in the mock translates to increases in Firmicutes representation in complex microbiome samples. Since the yield of extracted DNA varied according to the cell input amounts according to sample type and manufacturer’s instructions, and recommended input varied over 100-fold across mock microbiome and fecal material, individual DNA extractions were considered successful if the PCR resulted in sufficient amplicon for sequencing.

### 2.5. Novel ‘Rapid’ KOH Alkaline Based Protocol (K)

DNA was extracted using the DNA Purification and 16S rRNA Amplification Kit (Shoreline Biome, Farmington, CT, USA) as per the manufacturer’s instructions. Briefly, 1–3 mg of fecal material or mock microbiome was picked up on a 1 µL calibrated inoculating loop and dispersed into 50 µL lysis buffer by briefly twisting the loop, and 50 µL KOH solution was added. Samples were heated to 95 °C for 5 min and cooled until precipitate formed. The precipitate was pelleted, and the supernatant containing lysed DNA was added to 50 µL of a purification buffer. Samples were incubated at 50 °C to bind DNA to capture beads. Capture beads were washed 2× with 70% ethanol, and DNA was eluted in 40 µL TE. An amount of 160 µL TE was added to the diluted DNA for a final volume of 200 µL DNA. Diluted DNA (10 µL) was used in PCR for all amplicons.

### 2.6. New Bead Pasting Protocol (B)

Samples were processed according to the manufacturer’s instructions using the Bead Beater Lysis Kit (Shoreline Biome). Cell pellets or 1–3 mg fecal samples were suspended in a 50 µL bead beater buffer, and a 15 µL aliquot was added to the tube containing the bead mixture. The sample was subjected to bead beating for 60 s, after which the sample was diluted in 100 µL TE. The sample was vortexed briefly, and the supernatant containing the DNA was transferred to a purification buffer. After a 5 min incubation, the beads containing DNA were captured by a magnet and washed 2× with 70% ethanol. DNA was eluted from capture beads in 40 µL TE. The sample was further diluted 1:5 in TE prior to use in PCR.

### 2.7. MasterPure Complete DNA and RNA Purification Kit (E)

DNA was extracted using the MasterPure Complete DNA and RNA Purification Kit (Epicentre) as per the manufacturer’s instructions. The provided 50 µg/µL proteinase K and tissue and cell lysis solution was mixed with bacterial cells and incubated at 65 °C for 15 min. Cell debris was pelleted by centrifugation, followed by DNA precipitation using the provided MPC Protein Precipitation Reagent. DNA was pelleted by centrifugation and rinsed with isopropanol and 70% ethanol. DNA was resuspended in TE buffer.

### 2.8. Qiagen PowerSoil Kit (HMP)

This protocol has been used to process stool samples for the HMP [15] and yielded strain-level microbiome taxonomic resolution for a few genera [16]. As per the HMP recommended protocol, samples were pre-treated at 65 °C for 10 min and then at 95 °C for 10 min. After pre-treatment, samples were processed as per the manufacturer’s instructions. Briefly, the pre-heated fecal suspension was transferred to PowerBead Tubes and mixed with C1 lysis solution. Bacterial cells were subjected to mechanical bead beating using a MO BIO Vortex Adapter tube holder for 10 min, after which cell debris was pelleted by centrifugation. Proteins were precipitated from the supernatant by mixing and incubating with buffer C2 at 4 °C and pelleted by centrifugation. Supernatants were next incubated with buffer C3 at 4 °C to remove inhibitors. Column binding buffer C4 was added, and supernatants were applied to spin columns and centrifuged to bind DNA. The columns were washed by centrifugation with buffer C5 and eluted by centrifugation in buffer C6.

### 2.9. QIAamp DNA Stool Kit (Q)

DNA was extracted using the QIAamp DNA Stool Kit as per the manufacturer’s instructions. The provided lysis butter ASL was mixed with samples, vortexed to suspend cells, and incubated at 70 °C for 5 min. Debris was pelleted by centrifugation. InhibitEX tablets provided with the kit were incubated with samples, and debris was pelleted by centrifugation. Proteinase K was added to the supernatant, and after 10 min incubation at 70 °C, samples were centrifuged to pellet debris. The lysate was applied to spin columns, and the columns were washed by centrifugation with AW1 and then by AW2. Following an additional spin to remove residual buffer, DNA was eluted by centrifugation in buffer AE (elution buffer) provided in the kit.

### 2.10. ZymoBIOMICS^TM^ DNA/RNA Mini Kit (Z)

The standard procedure recommended by ZymoBIOMICS was followed. Briefly, bacterial cells were lysed by bead beating using a DNA/RNA Shield Lysis Tube, followed by centrifugation to pellet debris. The supernatant was mixed with lysis buffer, transferred to a spin column, and centrifuged. Bound DNA was washed by centrifugation once with DNA/RNA Prep Buffer and twice with DNA/RNA Wash Buffer. DNA was eluted by centrifugation with DNase/RNase-free water provided in the kit. A Zymo-Spin III-HRC Filter was prepared by centrifugation with HRC Prep Solution, the eluted DNA was transferred to the filter and recovered by centrifugation. Eluted DNA was used in PCR.

### 2.11. Library Construction and 16S rRNA Amplicon-Based Sequencing

Three different amplicons from different regions of the 16S rRNA gene were prepared from bacterial DNA purified from custom mock and human fecal samples. Amplicons ranged in size from the 292 base V4 region, the 526 base V1V3 region, and the 1506 base V1V9 amplicon that included all variable regions of the 16S rRNA gene (Figure S1). PCR was performed as per the manufacturer’s instructions (Shoreline Biome). Briefly, 10 μL of extracted DNA (2 ng/μL) from each sample was added to the 96-well plate containing dried PCR primers with barcodes. An amount of 10 µL 2× PCR mix was added to each well. PCR conditions were unique to each amplicon, as described in Table S2. Primer sequences for V1V3, V4, and V1V9 amplicons that were used for PCR in custom mock experiments are detailed in Table S1. Amplicons V1V3 and V4 were sequenced on the MiSeq (Illumina), and V1V9 was sequenced on the Sequel (Pacific Bioscience) instrument.

### 2.12. Sequence Pre-Processing and Classification

V1V3 and V4 sequence reads generated using the Illumina platform were processed by removing the sequences with low quality (average qual < 25) and ambiguous codons (N’s). Chimeric amplicons were removed using UChime software (Ver 6.0.307). Sequence reads were analyzed using the mothur phylotype pipeline SOP [17] by direct classification. V1V9 sequencing reads were generated using the PacBio platform. Circular consensus (ccs) reads were created from the raw reads using PacBio software (Ver 3.4.0) with standard cutoffs of 3 passes and a minimum of 90% accuracy. The mean number of passes was much higher than 3 for most reads because the amplicons were less than ~1500 bases, so the resulting base calling accuracy was generally over 99.9%. PacBio sequences were demultiplexed and classified using SBanalyzer (Ver 2.2-3), a GUI-based software package. A custom algorithm was used to identify sample-specific DNA barcodes on each read, trim barcodes from each read, and sort the reads into individual files based on the barcodes. A custom reference database called ‘Athena’ was built for classification, which contains contiguous 16S–23S gene regions from organisms sequenced and assembled such that individual 16S genes could be positioned in the assembly. The mapping algorithm was based on BLASTplus v2.8.0 (ftp://ftp.ncbi.nlm.nih.gov/blast/executables/blast+/LATEST/ (accessed on 13 December 2018)). The mock community was analyzed at the species level, and human stool samples were analyzed at the genus level.

### 2.13. Statistical Analyses

Differences between lysis methods HMP and K applied to human stool samples were tested and evaluated using the paired *t*-test for alpha-diversity and Analysis of Molecular Variance (AMOVA) for beta-diversity analyses. Differentially abundant taxa depicted in heatmaps and significant driver taxa in non-metric multidimensional scaling (NMDS) plots were selected using the Wilcoxon Signed Rank test based on their relative abundances. The Benjamini–Hochberg false discovery rate method was used for multiple comparison adjustments.

### 2.14. Data Sharing

Sequences were submitted to the Sequence Reads Archive (PRJNA531279).

## 3. Results

### 3.1. Evaluation of Six DNA Extraction Methods Using a Mock Community Containing 10 Bacterial Species

**Figure 1:**
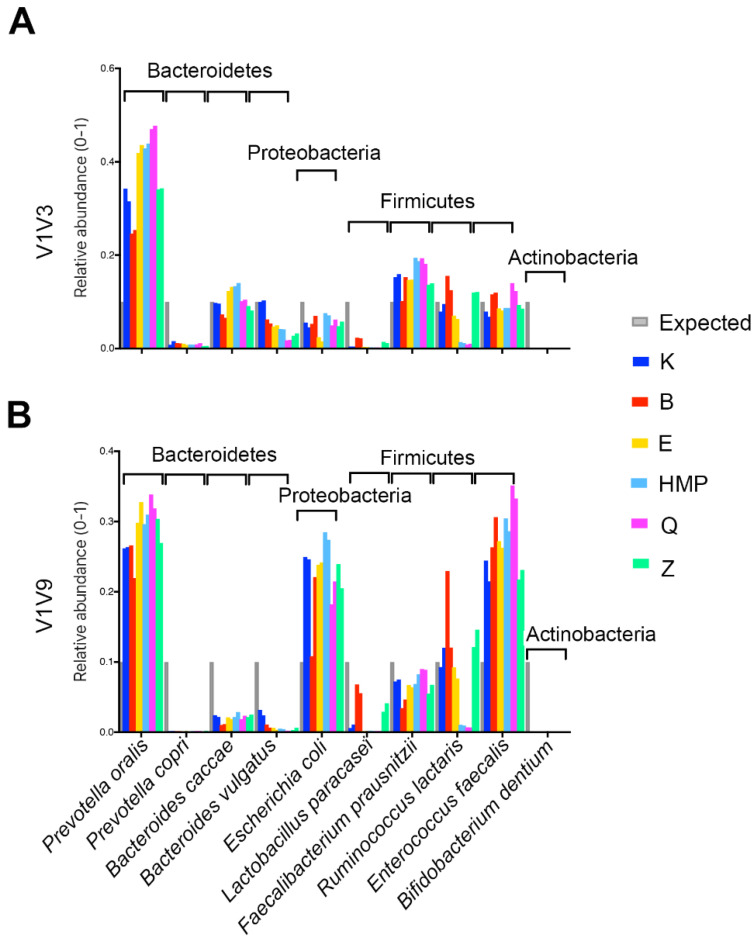
Evaluation of six DNA extraction methods using a mock community containing 10 bacterial species. Six different lysis methods were used to prepare DNA from the 10 mock species, in duplicate; 10 mock species are shown on the *x*-axis using V1V3 (**A**) and V1V9 (**B**) of hypervariable regions in the 16S rRNA gene. The relative abundance of each organism is shown on the *y*-axis. The grey bar at the left of each organism displays the expected relative abundance of 16S rRNA copies based on the normalized input cell number for each bacterium. Novel K and B methods demonstrated equal or improved accuracy and are therefore less biased results compared to the 4 commercially available, widely used protocols (E, HMP, Q, and Z).

Six lysis methods listed in Table 1 were evaluated using a custom mock microbiome consisting of the 10 bacterial species listed in Table S2. For all lysis methods, the DNA extraction protocol was followed by amplification of two 16S rRNA gene regions, one containing regions V1V3 and one covering the full-length V1V9 16S rRNA gene (Figure 1). The different amplicon regions were compared for the ability to capture diversity and facilitate taxonomic identification. Reads were rarified to 4145 reads per sample for V1V3 and 5156 reads per sample for V1V9 amplicons.

Analysis of multiple amplicon regions of the 16S rRNA gene provided useful platforms for differentiating lysis and DNA preparation bias from PCR and sequencing bias across six lysis methods tested. Duplicate DNA preparations were generally consistent for each duplicate sample preparation. Of the six methods tested, novel K and B methods performed most consistently, followed closely by the Z method. The most striking differences across the six methods were observed in Firmicutes species. There were two methods, HMP and Q, that performed poorly for the Firmicutes *R. lactaris* regardless of the amplicon region tested. The E, HMP, and Q methods yielded lower representation for *L. paracasei* for both amplicons as compared to the K and B methods. There are consistent biases that appear to be related to sequencing or amplicon efficiency. For example, *P. copri* was consistently under-represented across all six methods, and there were no reads recovered for *B. dentium* for any of the methods. Representation of *Bifidobacterium* is strongly dependent on the V1 primer sequence and the V1 primer sequence detailed in Table 1 shows variation at three bases in the primer sequence, perhaps explaining the lack of *Bifidobacterium* reads. Other mock species representations such as *B. caccae*, *E. coli*, *E. faecalis*, *P. oralis,* and *F. prausnitzii* were more or less similar across the six lysis methods tested. It should be noted that *E. coli* has one base at position 12 in the V1 site that is different from the V1 primer, which could negatively affect PCR efficiency (Table 1). *F. prausnitzii* also has a single base change in the V3 primer, which did not seem to inhibit representation.

The custom mock microbiome results demonstrate that the novel K and B methods compare favorably to some of the most widely used methods (E, HMP, Q, and Z), with the added benefit that the K method reduces hands-on time by up to 20× for 96 sample preparations. Results from a commercial mock community containing eight bacteria confirmed the effectiveness of the K protocol on a second mock microbiome (Figure S2). The published bead beating data from the Z kit manufacturer was compared to the new K method data. The results were similar even though the V4 16S region PCR conditions and primer sets were different, different lots of D-6300 mock community were used, and the results were obtained from different sequencing runs performed at different sites, demonstrating that the consistency observed is robust and reproducible.

### 3.2. Evaluation of Number of Sequences Reads for Community Structure

Further testing was conducted on human fecal samples to determine whether the increase in representation observed for specific Firmicutes bacteria in the custom mock microbiome translates into increased recovery of a variety of Firmicutes from 20 different samples in a complex human fecal sample matrix. A direct comparison of the K protocol and the standard HMP protocol was conducted on stool samples from cancer patients and matched controls.

One of the most important considerations in any microbiome experiment is the minimum number of reads required for a valid view of the representation of diversity. An understanding of the minimum number of reads per sample is needed to set the reads/sample threshold in experimental design, which drives the amount of sequencing needed to arrive at meaningful results. The requirement of reads per sample can be one of the major costs associated with microbiome experiments. Since the target reads/samples are set during the sequencing step, prior to analysis, a poor decision can result in too few reads to obtain meaningful comparisons between samples, or too many reads, which increases cost per sample. Since the different sequencing technologies such as Illumina and PacBio used in the present study offer a wide variation in the number of sequences per run, and multiplexing can be used to analyze a large number of samples per run, an analysis was performed to determine the minimum number of reads (read sample size), which supports a robust determination of taxonomic diversity. Figure S3 demonstrates that the number of reads from the fecal sample dataset could be decreased to 1100 while maintaining the ability to accurately describe community structure. However, it is clear that more than 500 reads per sample are needed for consistent relationships between sample groups. After careful examination of minimum sequence reads to maintain the majority of community structure in the present study, sequencing reads were rarified at 2538 and 1057 for V1V3 and V1V9 amplicons, respectively. Rarefication based on tree clustering community structure analysis resulted in 20 and 11 subjects remaining for V1V3 and V1V9 amplicons, respectively, in our data.

### 3.3. Significant Firmicutes/Bacteroidetes Lysis Differences for Fecal Microbiome Cancer Samples and Controls

**Figure 2:**
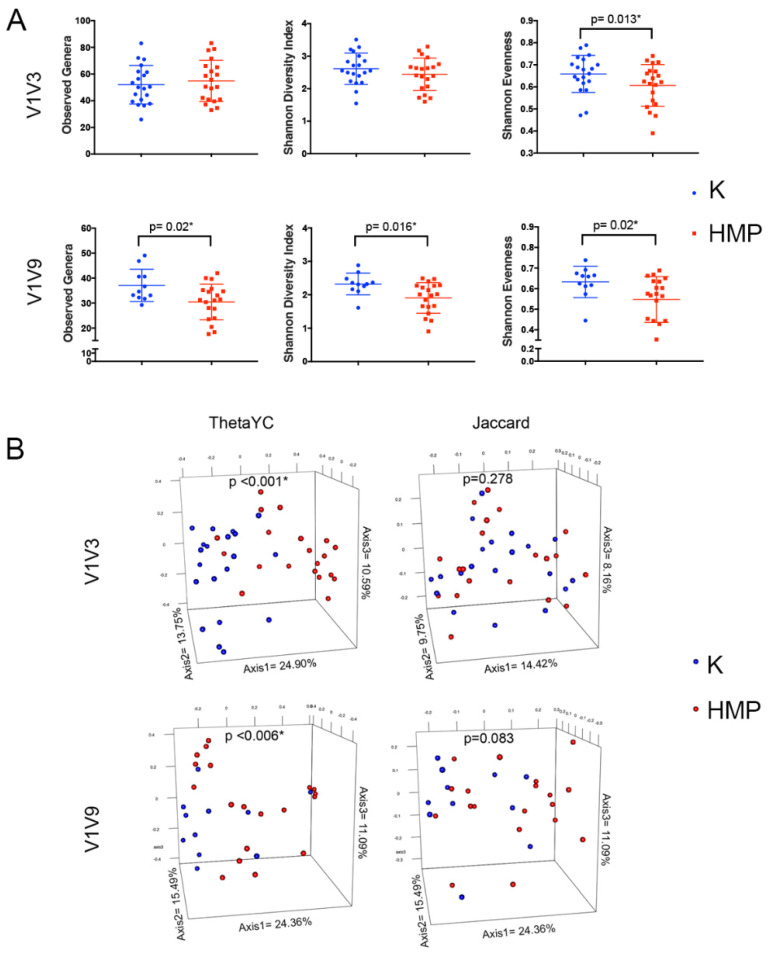
Comparison of the lysis method on the microbial diversity of human fecal samples. Twenty fecal samples were lysed, and DNA was prepared using methods ‘K’ (blue) and ‘HMP’ (red), 16S amplicons V1V3 and V1V9 were sequenced, and microbial diversity was analyzed in panels (**A**) and (**B**). In panel (**A**), for each sample, the number of observed genera, Shannon Diversity, and Shannon Evenness were plotted. V1V3 sequencing demonstrated significantly increased evenness using the K method, whereas the number of genera and Shannon Diversity were similar (above *p* = 0.05) for the samples from both methods. V1V9 sequencing demonstrated significant increases in observed genera richness and Shannon Diversity and Evenness of bacterial communities using the K method. In panel (**B**), ThetaYC and Jaccard distance-based PCoA plots are shown. Jaccard’s index calculates the ratio of the number of shared species as compared to the number of distinct species. ThetaYC is a measure of dissimilarity between the structures of two communities that includes species proportions of both the shared and non-shared species in each population. ThetaYC showed significant bacterial community structure differences for the samples prepared using both K and HMP methods, but differences were not seen in the Jaccard plots.

To explore how the K and HMP lysis methods and amplicon length affect bacterial population information recovered from fecal samples, alpha diversity was calculated by comparing protocols and amplicons (Figure 2A). For the full-length 16S V1V9 amplicon, the ‘K’ method significantly increased the number of taxa identified (*p* = 0.02), diversity (*p* = 0.016), and evenness (*p* = 0.02), indicating that the ‘K’ method’s effect on lysis had consequences for downstream analyses. A similar analysis of the V1V3 amplicon showed that evenness was improved (*p* = 0.013) using the K method, indicating a better lysis of additional bacterial cells, while there was no statistical difference in richness or diversity between methods. The difference in richness and diversity between the amplicons may be attributed to the difference in the ability of the different-sized amplicons to differentiate between taxa. The full 1500 bp 16S rRNA gene is capable of better differentiation of bacterial taxonomy than the 525 bp V1V3 amplicon because of the 1000 bases of extra sequence. As a result, the longer amplicon is likely to be able to differentiate a higher number of closely related taxa that appear because of lysis improvements. On the other hand, no difference in richness and diversity between K and HMP protocols in V1V3 amplicons can be partially due to the differences in sequencing depth. In Figure 2B, principal coordinate analysis (PCoA) showed that community structure was significantly different (*p* < 0.001, *p* < 0.006) between the K and HMP methods when using the ThetaYC distance, which takes into account both the composition of species and the relative proportions of those species. Combined with evidence shown in Figure 2A, Figure 2B indicates that the K method extracts significantly more bacterial cells at the level that the diversity of the bacterial community was significantly increased using the K method. Next, we investigated whether the K method finds additional taxa that were absent from using the HMP method. Using Jaccard distance-based community composition which measures only the membership, not relative abundance, did not show a significant difference between the two methods (Figure 2B). This was our key evidence that shows the K method lyses the same members of bacterial taxa while significantly increasing those taxa compared to the HMP method. These results are consistent with the hypothesis that the K method lyses significantly more bacterial cells that might be harder to be lysed sufficiently using the HMP method in abundance. The analysis of the alpha and beta diversity results for the V1V3 and V1V9 regions demonstrated that there is a potential for increased lysis of Firmicutes species using the K method. This translated into an increase in observed sample diversity, rather than just a shift in representation. The results are also consistent with the idea that longer amplicons with increased taxonomic representation combined with improved lysis offer the opportunity for increased diversity at lower read levels, which reflects the true bacterial community.

**Figure 3:**
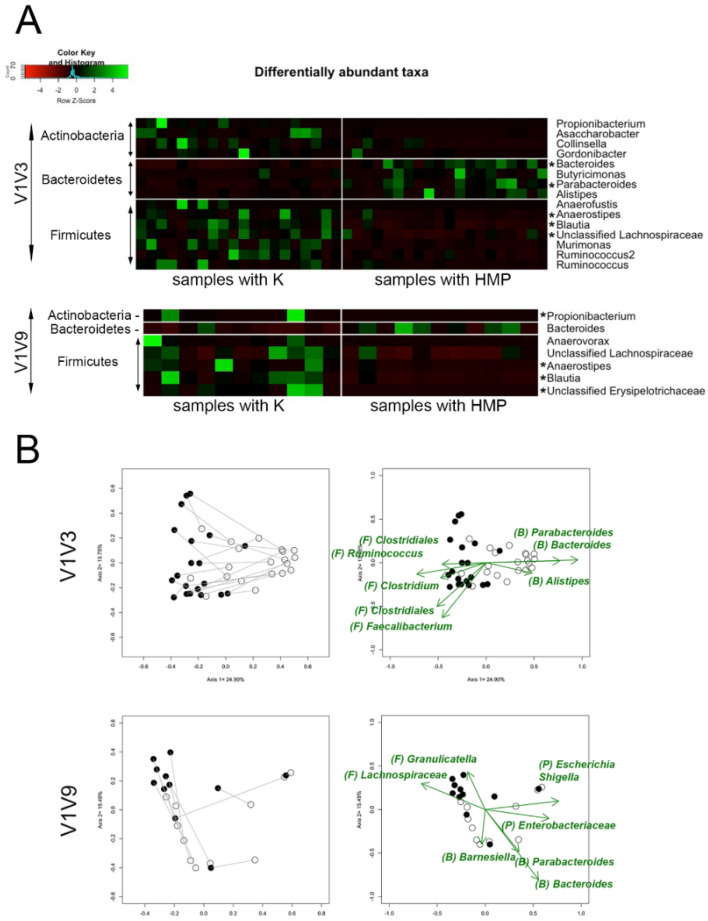
Individual taxon differences between K and HMP protocols using human stool samples. In panel (**A**), a heatmap displays taxa that varied significantly with the lysis method, using the z-score from relative abundances of each taxon; 15 taxa (V1V3, Illumina) and 7 taxa (V1V9, PacBio) were significantly different. Significant genera remaining after multiple comparison adjustment calculations were labeled with an Asterix. In panel (**B**), NMDS plots show specific driver genera that varied significantly by lysis methods K and HMP. Filled circles designate samples processed using the K method, while empty circles correspond to the HMP method. Individual stool samples have a line connecting the result for the different methods for that sample. Driver genera (green arrows) are driving the differences between results obtained from the ‘K’ and ‘HMP’ methods. Phyla were abbreviated (P) = Proteobacteria, (B) = Bacteroidetes, (F) = Firmicutes.

To address the hypothesis that Firmicutes representation in fecal samples is dependent on the lysis method, we determined which taxa showed relative abundance differences between K and HMP methods in the 20 fecal samples. The V1V3 amplicon showed a consistent shift in community structure that was dependent on the K and HMP methods. Firmicutes and Actinobacteria species were significantly increased in samples lysed with the K protocol, while Bacteroidetes species were significantly increased in samples lysed with the HMP protocol (Figure 3A). This result for fecal samples corresponds with the custom mock microbiome evidence that the K protocol is a more effective lysis method for Gram-positive bacteria, recovering a more representative microbiome profile from stool samples as well as mock samples. The V1V9 results demonstrate that the trend is consistent across two amplicons and Illumina and PacBio sequencing platforms. Figure 3B visualizes the lysis-dependent shift in specific taxa using NMDS plots, an independent method for visualizing whether taxa are affected by the lysis method. Several Firmicutes genera such as *Clostridiales genus*, *Ruminococcus*, *Clostridium*, and *Faecalibacterium* were the significant drivers of communities that shifted in a negative direction on axis 1, where most of the ‘K’ method samples clustered. In contrast, the ‘HMP’ method stool samples tended to cluster in the positive direction on axis 1, and Bacteroidetes genera such as *Parabacteroides*, *Bacteroides*, and *Alistipes* were the significant drivers. This trend was consistent across the 11 samples with sufficient V1V9 amplicon data, where there was a significant correlation between the ‘K’ method and increases in Firmicutes genera such as *Lachnospiraceae genus and Granulicatella*, whereas the ‘HMP’ method correlated with increased representation of Bacteroidetes, as well as Proteobacteria genera. Significantly higher levels of Firmicutes were the driver for axis shifts for almost all samples prepared using the ‘K’ method, which is consistent with the hypothesis that method ‘K’ lyses Firmicutes that are under-represented by the ‘HMP’ method. Simultaneous increases in Firmicutes and reduction of Bacteroidetes representation are likely because the 16S data are relative abundances, where an increase in a specific taxon will result in decreases of other taxa. In summary, the taxa responsible for the changes in representation in fecal samples parallel what was seen for the mock microbiome samples, where the ‘K’ method improved Firmicutes representation as compared to the ‘HMP’ method.

## 4. Discussion

In this study, we benchmark a novel ‘rapid’ alkaline microbiome DNA extraction procedure ‘K’ that does not contain bead beating or enzymatic methods to assess suitability for 16S rRNA gene or other amplicon-based microbiome survey applications. 

Bead beating is a time-consuming process that is not easily automated, can vary in effectiveness depending on how it is implemented, and has the potential to degrade the DNA of the easily lysed organisms while missing DNA from more difficult Gram-positive bacteria. Digestion with proteases and/or lysozyme adds time, complexity, and variability to DNA preparation methods because bacteria can vary widely in susceptibility to enzymatic digestion. Effective lysis methods that avoid bead beating and enzymatic steps have the potential to eliminate costs associated with bead beater hardware, reagents and disposables, and enzyme costs and reduce lysis variability, decrease time to result, and enable high throughput handling and/or automation.

A comparison of multiple commercial methods and a commonly used ‘HMP’ method to the novel ‘K’ method clearly demonstrated that bead beating and enzymatic digestion are not sufficient for comprehensive bacterial lysis. In fact, the ‘K’ method improved the recovery of Gram-positive Firmicutes as compared to multiple commercial methods for both a custom mock community as well as human fecal samples. Although mock communities can be useful for assessing lysis effectiveness, it can be difficult to determine whether any given lysis method is universally effective using a mock, because not all microbes can be present in a mock microbiome, and the susceptibility of different strains to lysis conditions can vary. For example, method ‘K’ yielded results similar to both the expected profile and the manufacturer’s optimized protocol for a commercially available mock community based on the proportions of microbes measured using the V4 16S rRNA gene amplicon and Illumina sequencing. However, it was apparent from similar tests with V1V3 and V1V9 amplicon that the choice of PCR primers and amplicon, lysis methods, and bacteria selected for the mock community can be sources of variability in the microbial DNA profile. To isolate and study the effects of lysis methods, the relative performance of multiple lysis and DNA purification methods was assessed by sequencing the purified DNA from a custom mock community and fecal samples using multiple PCR amplicons and sequencing technologies. A custom mock microbiome containing selected Gram-positive Firmicutes strains that were known to be difficult to lyse demonstrated that the ‘K’ method provided an improved representation of Firmicutes. Additional comparisons of the ‘K’ and ‘HMP’ methods on twenty human fecal samples demonstrated that the microbial profiles of specific bacteria depended on the lysis method and that the ‘K’ method yielded improved representation of multiple Firmicutes, which is in agreement with the custom mock community results. Representation of certain Actinobacteria species was also improved by the ‘K’ method, demonstrating that not all difficult-to-lyse bacteria are Firmicutes.

Furthermore, 16S rRNA amplicon-based sequencing profiling of microbial populations remains popular because it is cost-effective, and taxonomic identification of sequencing reads can be facilitated by multiple well-understood data analysis tools. These tools enable rapid mapping of sequenced data against large datasets such as the RDP [18], SILVA [19], GreenGenes [20], EasyTaxon [21], and Athena databases used in the present study. It should be possible to extend this methodology to other amplicon-based targets such as fungi and other non-bacterial organisms in the community. Thus, a rapid, comprehensive lysis solution for amplicon-based analysis is a useful addition to reduce costs, variability, and time to result in microbiome profiling.

Lysis and DNA purification is arguably one of the most important steps in sequencing-based microbiome profiling because bacteria need to be lysed to release the DNA for sequencing. Unfortunately, lysis is also one of the most variable steps in microbiome profiling, because minor variations in standard methodologies can result in under-treatment (un-lysed organisms) or over-treatment (DNA damage). Contributions to the variability of lysis include differential resistance of cell wall and cell membrane structures to lysis methods such as protease and lysozyme treatment, as well as variability inherent in physical processes like bead beating. In order to determine which protocols provide the best DNA extraction outcomes, where the DNA recovered from bacterial lysis closely represents the original bacterial community, several different DNA extraction methods that employ lysozyme [22], proteinase K [23], physical disruption methods such as bead beating [15] and temperature change [24], chemicals [25], or combinations of each [26] were tested. Lysozymes can be used to target bacterial cell walls, but different lysozymes have different effectiveness against the variety of bacterial cell wall chemistries and layers of phospholipid, peptidoglycan, and protein that bacteria employ. Physical disruption can promote lysis of Gram-positive bacteria with tougher cell walls, but bead beating suffers from sample-to-sample variability, is not easily scalable for high throughput sample processing or automation, damages DNA [27], and negatively affects recovery from low-input samples. It can be difficult to standardize results across different microbiome investigations because each sample type requires independently optimized DNA extraction methods based on the sample type for optimal yield [1,28]. In this report, we assess different methods, including an alternative alkaline DNA extraction method for 16S rRNA amplicon sequencing strategies that were designed to eliminate variable processes such as bead beating and enzyme treatment, increase lysis efficiency for difficult bacteria, and enable high throughput applications by reducing the number of steps, decreasing handling time, and improving efficiency.

In summary, we benchmark a novel K DNA extraction protocol that avoids bead beating and enzymatic treatments, while at the same time demonstrating improved performance compared to commonly used DNA lysis and purification methods for the accurate representation of mock communities and human fecal gut microbiome samples. We conclude that the novel ‘K’ DNA extraction protocol offers a reliable alternative for preparing fecal specimens for 16S rRNA gene amplicon sequencing that maintains the representation of microbial populations in a sample.

## Figures and Tables

**Table 1:** Summary of 6 different DNA extraction protocols used in this study.

ID	Kit Name	Sample Used in This Study	Bead Beating Based	Enzyme Based	Time 96 Samples	Note
K	novel ‘Rapid’ KOH protocol protocol (Intus Biosciences, Farmington, CT, USA)	Mock and Stool	no	no	44 min	ssDNA
B	Bead pasting	Mock	yes	no	80 min	
E	MasterPure Complete DNA and RNA Purification Kit (LGC Biosearch Technologies, Hoddesdon, UK)	Mock	no	yes	400 min	
HMP	Qiagen PowerSoil kit (Qiagen, Germantown, MD, USA)	Mock and Stool	yes	no	400 min	HMP protocol (Heating added)
Q	QIAamp DNA Stool Kit (Qiagen, Germantown, MD, USA)	Mock	no	yes	320 min	
Z	ZymoBIOMICS^TM^ DNA/RNA Mini Kit (Zymo Research Corporation, Irvine, CA, USA)	Mock	yes	no	280 min	

## Supplementary Materials

The following supporting information can be downloaded at: https://www.mdpi.com/article/10.3390/ijms25052966/s1.
