# Supplementary material for: Improved DNA Extraction and Amplification Strategy for 16S rRNA Gene Amplicon-Based Microbiome Studies
Source: Int J Mol Sci. 2024 Mar 4;25(5):2966. doi: 10.3390/ijms25052966 (PMC10932036; doi:10.3390/ijms25052966)
Supplement: Supplementary file 1 [file ijms-25-02966-s001.zip › ijms-2890713-supplementary.pdf]

**Table S1** Custom mock microbiome organisms with corresponding genomic sequences and primers from target 16S rRNA gene regions. Black bases match the most common consensus sequence. Green bases signify less common variants that are included in the primer degeneracy by design. Red bases identify mismatches between the primer sequence and the genomic DNA that were not included in the primer design. Symbols for degenerate base positions are listed.

| ReadID | V1 Forward | V3 Reverse | V4 Forward | V4 Reverse | V9 Reverse |
|---|---|---|---|---|---|
| *Lactobacillus paracasei* | AGAGTTTGATCCTGGCTCAG | ATTACCGCGGCTGCTGG | na | na | TACGGCTACCTTGTTACGACTT |
| *Enterococcus faecalis* | AGAGTTTGATCCTGGCTCAG | ATTACCGCGGCTGCTGG | na | na | TACGGCTACCTTGTTACGACTT |
| *Bacteroides vulgatus ATCC 8482* | AGAGTTTGATCCTGGCTCAG | ATTACCGCGGCTGCTGG | na | na | TACGGCTACCTTGTTACGACTT |
| *Prevotella oralis* | AGAGTTTGATCCTGGCTCAG | ATTACCGCGGCTGCTGG | na | na | TACGGCTACCTTGTTACGACTT |
| *Bacteroides caccae* | AGAGTTTGATCCTGGCTCAG | ATTACCGCGGCTGCTGG | na | na | TACGGCTACCTTGTTACGACTT |
| *Prevotella copri* | AGAGTTTGATCCTGGCTCAG | ATTACCGCGGCTGCTGG | na | na | TACGGCTACCTTGTTACGACTT |
| *Faecalibacterium prausnitzii* | AGAGTTTGATCCTGGCTCAG | **T**TTACCGCGGCTGCTGG | na | na | TACGGCTACCTTGTTACGACTT |
| *Ruminococcus lactaris* | AGAGTTTGATCCTGGCTCAG | ATTACCGCGGCTGCTGG | na | na | TACGGCTACCTTGTTACGACTT |
| *Escherichia coli str. K-12* | AGAGTTTGATC**A**TGGCTCAG | ATTACCGCGGCTGCTGG | na | na | TACGG**T**TACCTTGTTACGACTT |
| *Bifidobacterium dentium* | AG**G**GTT**C**GAT**T**CTGGCTCAG | ATTACCGCGGCTGCTGG | na | na | TACGGCTACCTTGTTACGACTT |
| *Pseudomonas aeruginosa* | na | na | GTGCCAGCAGCCGCGGTAA | ATTAGATACCCTGGTAGTCC | na |
| *Escherichia coli* | na | na | GTGCCAGCAGCCGCGGTAA | ATTAGATACCCTGGTAGTCC | na |
| *Salmonella enterica* | na | na | GTGCCAGCAGCCGCGGTAA | ATTAGATACCCTGGTAGTCC | na |
| *Listeria monocytogenes* | na | na | GTGCCAGCAGCCGCGGTAA | ATTAGATACCCTGGTAGTCC | na |
| *Bacillus subtilis* | na | na | GTGCCAGCAGCCGCGGTAA | ATTAGATACCCTGGTAGTCC | na |
| *Lactobacillus fermentum* | na | na | GTGCCAGCAGCCGCGGTAA | ATTAGATACCCTGGTAGTCC | na |
| *Staphylococcus aureus* | na | na | GTGCCAGCAGCCGCGGTAA | ATTAGATACCCTGGTAGTCC | na |
| *Enterococcus faecalis* | na | na | GTGCCAGCAGCCGCGGTAA | ATTAGATACCCTGGTAGTCC | na |
| **Seqeunces for V1V9 amplicon** | AGAGTTTGATC**M**TGGCTCAG | | | | TACGG**Y**TACCTTGTTACGACTT |
| **Seqeunces for V1V3 amplicon** | AGAGTTTGATCCTGGCTCAG | ATTACCGCGGCTGCTGG | | | |
| **Seqeunces for V4 amplicon** | | **W**TTACCGCGGCTGCTGG | GTG**Y**CAGC**M**G**YY**GCGGT**R**A | GGA**Y**TAC**NV**GGGT**H**TCTAA | |
| **Amplicon Size** | | **~556 bp V1V3** | | **~253bp V4** | **~1550 bp V1V9** |
| enomic Target Sequences without exact primer match | | | | | |
| Green Base= degenerate primer sequence | | | | | |
| Red base = Mismatch from Consensus | | | | | |
| Base Symbols: M=A/C Y=C/T W=A/T | | | | | |

**Table S2** Summary of 16S rRNA amplification protocols for V1V3, V4, and V1V9 used in this study.

| | V1-V3 | | V4 | | V1-V9 | |
|---|---|---|---|---|---|---|
| Initial Denaturation | 95°C, 3:00 | X 1 | 95°C, 3:00 | X 1 | 95°C, 3:00 | X 1 |
| Denature | 95°C, 0:10 | X 32 | 95°C, 0:20 | X 34 | 95°C, 0:30 | X34 |
| Anneal | 57°C, 0:10 (Ramp speed 0.5°C/sec) | | 58°C, 0:15 (Ramp speed 4°C/sec) | | 63°C, 0:45 (Ramp speed 4°C/sec) | |
| Extend | 73°C, 0:21 | | 72°C, 0:15 | | 72°C, 1:30 | |
| Final Extension | 73°C, 0:45 | X 1 | 72°C, 2:00 | X 1 | 72°C, 3:00 | X 1 |

Supplementary figure legend

**Figure S1. Amplicons used in the Study**. The positions of the V4, V1V3, and V1V9 amplicons in the 16S rRNA gene are represented by labeled green, red, and blue boxes, respectively. The 16S rRNA gene is shown with respect to approximate placement of other tRNA and rRNA genes as found in many bacterial genomes. The approximate base position of 16S rRNA gene sequences used as amplicon primers are shown, as 27F, 513F, 806R, 513R, 1492R.

**Figure S2. K Protocol Compared to Z Bead Beating Protocol Using a Commercial Mock Microbiome.** Two sample replicates of bacterial cells from the ZymoBiomics D-6300 mock community standard was lysed using the Shoreline Biome rapid 'K' method followed by V4 16S rRNA gene sequencing. Z results shown were included as reported from V3-V4 amplicon after 1 minute processing using the Precellys Evolution tissue homogenizer, followed by DNA extraction using the ZymoBiomics DNA Miniprep Kit. The Zymo application note 'Use of the Precellys® Evolution Homogenizer for Unbiased DNA Extraction from the Zymobiomics® Microbial Community Standard' https://files.zymoresearch.com/datasheets/bertin_-_zymo_research_-_zymobiomicsmicrobialcommunitystandard_-_app_note.pdf.

**Figure S3**. **Minimum Number of Reads Needed Per Sample.** A minimum of 1100 reads per sample was estimated to be sufficient for consistent taxonomic discrimination for fecal samples. Unrooted tree plots were created for each sample using 4155, 2538, 1100, 500, and 50 reads per sample. Closely related samples are shown by clustering in panel A, with closely related samples sharing the same color. Relationships between samples were compared for consistency as the number of reads was decreased. Close clustering of related sample colors was maintained for 4155, 2538 and 1100 reads/sample, but related groups start separating at 500 reads/sample.

Figure S1.

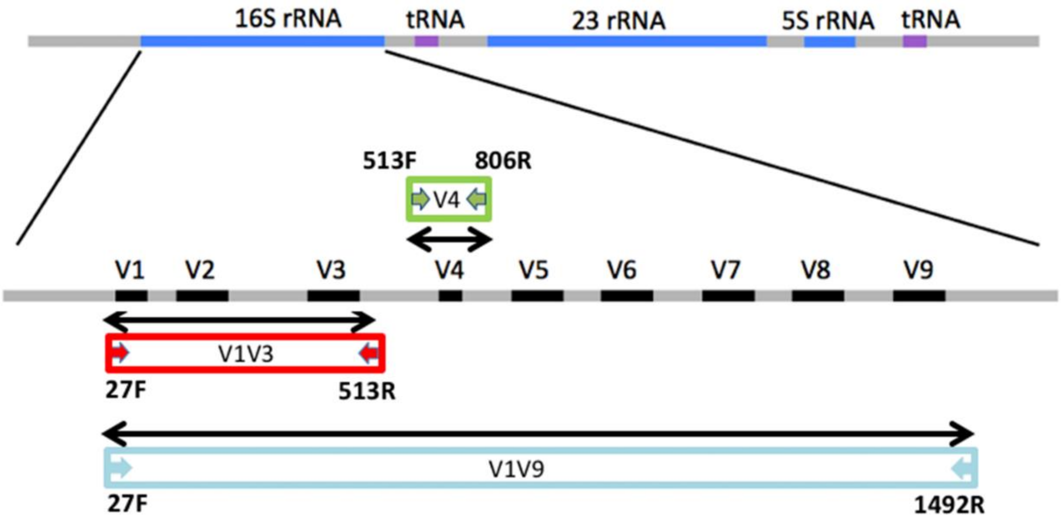

16S rRNA
tRNA
23 rRNA
5S rRNA
tRNA
513F
806R
V4
V1
V2
V3
V4
V5
V6
V7
V8
V9
V1V3
27F
513R
V1V9
27F
1492R

Figure S2.

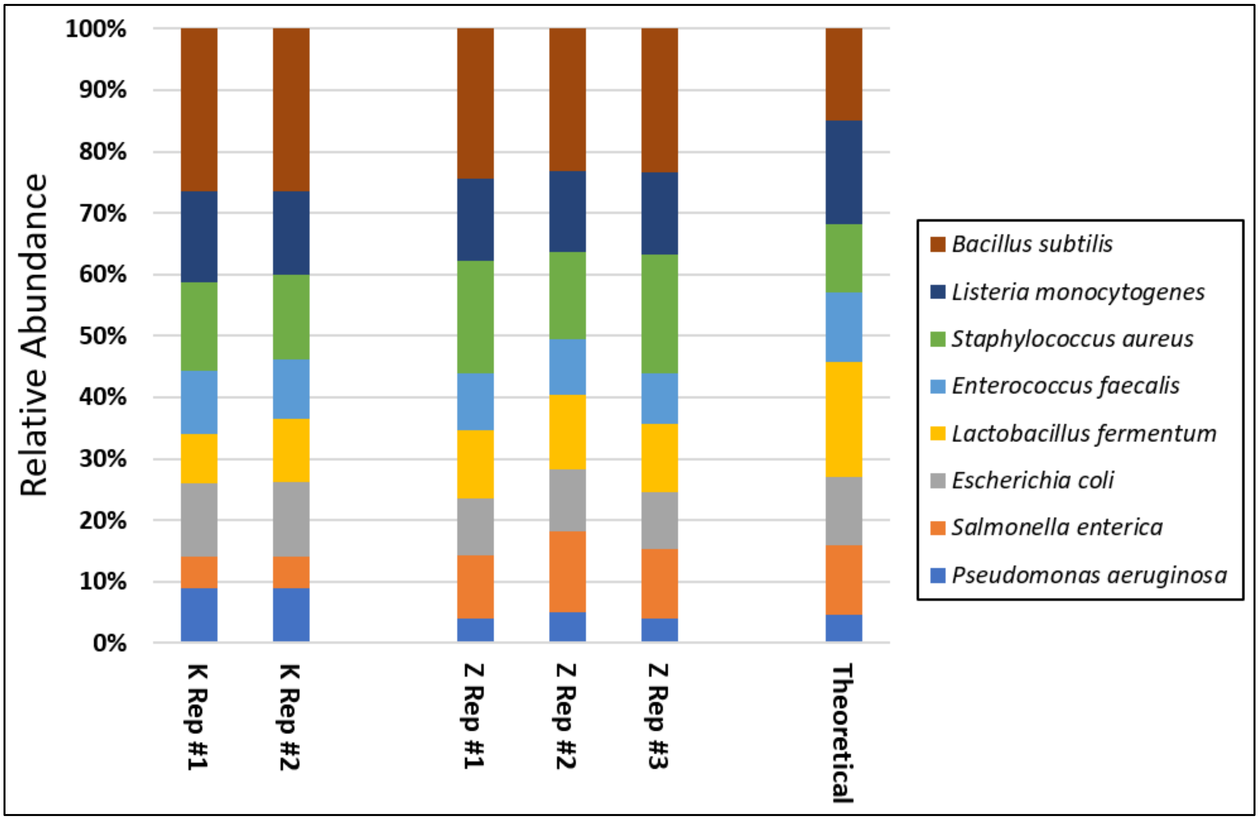

Relative Abundance
100%
90%
80%
70%
60%
50%
40%
30%
20%
10%
0%
K Rep #1
K Rep #2
Z Rep #1
Z Rep #2
Z Rep #3
Theoretical
Bacillus subtilis
Listeria monocytogenes
Staphylococcus aureus
Enterococcus faecalis
Lactobacillus fermentum
Escherichia coli
Salmonella enterica
Pseudomonas aeruginosa

Figure S3.

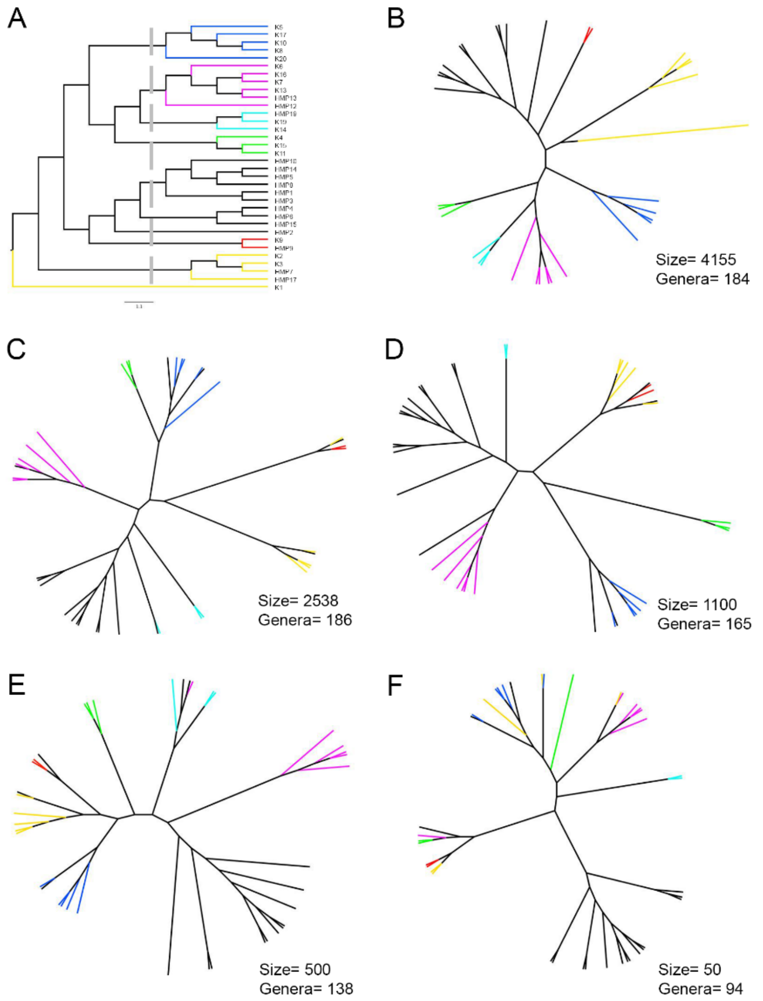
A
B
Size= 4155
Genera= 184
C
Size= 2538
Genera= 186
D
Size= 1100
Genera= 165
E
Size= 500
Genera= 138
F
Size= 50
Genera= 94
